# On the Effect of Practice on Exploration and Exploitation of Options and Strategies

**DOI:** 10.3389/fpsyg.2021.725690

**Published:** 2021-11-12

**Authors:** Doron Cohen, Kinneret Teodorescu

**Affiliations:** ^1^Center for Economic Psychology, University of Basel, Basel, Switzerland; ^2^Faculty of Industrial Engineering and Management, Technion, Haifa, Israel

**Keywords:** exploration and exploitation, practice, decisions from experience, Ill-structured problem, strategies, inertia, status quo

## Abstract

Insufficient exploration of one’s surroundings is at the root of many real-life problems, as demonstrated by many famous biases (e.g., the status quo bias, learned helplessness). The current work focuses on the emergence of this phenomenon at the strategy level: the tendency to under-explore the set of available choice strategies. We demonstrate that insufficient exploration of strategies can also manifest as excessive exploration between options. In such cases, interventions aimed at improving choices by reducing the costs of exploration of options are likely to fail. In Study 1, participants faced an exploration task that implies an infinite number of choice strategies and a small sub-set of (near) optimal solutions. We manipulated the amount of practice participants underwent during the first, shorter game and compared their performance in a second, longer game with an identical payoff structure. Our results show that regardless of the amount of practice, participants in all experimental groups tended to under-explore the strategy space and relied on a specific strategy that implied over-exploration of the option space. That is, under-exploration of strategies was manifested as over-exploration of options. In Study 2, we added a constraint that, on a subset of practice trials, forced participants to exploit familiar options. This manipulation almost doubled the per-trial average outcome on the comparable longer second game. This suggests that forcing participants to experience the effects of different (underexplored) strategy components during practice can greatly increase the chance they make better choices later on.

## Introduction

Many famous inertia biases such as the status quo bias (Samuelson and Zeckhauser, 1988), the sunk cost effect (Arkes and Blumer, 1985), the default effect (Johnson and Goldstein, 2003), learned helplessness (e.g., Teodorescu and Erev, 2014a) etc., reflect the tendency to repeat familiar and/or default options, even when better alternatives exist. That is, people tend to prefer known alternatives and to avoid the inherent risk and uncertainty embodied in exploration of new, potentially better, alternatives. These biases have been previously identified as major impediments for attempts to improve real life decisions (e.g., Samuelson and Zeckhauser, 1988). For example, Larcom et al. (2017) found that a major disruption of service in the London Underground system (due to a strike) significantly improved commuters route choices. This unexpected result was explained as a product of the strike interrupting inertia and forcing commuters to explore better routes. Other studies suggest inertia biases can lead to suboptimal pension and insurance choices (Johnson et al., 1993; Madrian and Shea, 2001), to medical non-compliance (Suri et al., 2013) and resistance to adopting new technologies (Kim and Kankanhalli, 2009; Polites and Karahanna, 2013).

Research suggests many of these biases can be understood as a problem of insufficient exploration of new options. For example, Teodorescu and Erev (2014a, b) focused on settings where exploration of new options is costly but advantageous as it can lead to the discovery of great rewards (i.e., a rare “Treasures” environment). In such settings, the researchers found that people tend to insufficiently explore due to the low prevalence of rewards from exploration. This implies that inertia biases arise when people under-explore different options (due to the low frequency of rewards exploration is perceived to yield) and thus better options (e.g., routes) are overlooked (see also Roth et al., 2016 for a similar observation regarding insufficient checking).

Thus, one natural solution to help people overcome sub-optimal inertia is to directly reduce both the perceived and the experienced costs of exploring new options. For example, Ashby and Teodorescu (2019) found that reducing the monetary costs of switching between alternatives reduced inertia biases in a binary choice setting. Similarly, reducing the costs of exploration between service providers, e.g., by enforcing transparency of prices and providing easy comparison tools, reduces information costs and tends to alleviate consumer’s inertia biases (e.g., see Timmons et al., 2019; Byrne et al., 2020; Marandola et al., 2020). In fact, encouraging switching between service providers by reducing the associated switching costs is generally understood as a major factor for improving consumer welfare (BEREC, 2010; Ofcom, 2010; Marandola et al., 2020; Heiss et al., 2021). For example, research suggests that ensuring hassle-free and cheap portability between cell-phone carriers (e.g., with regulation that effectively reduce portability costs, see BEREC, 2010; Sánchez and Asimakopoulos, 2012) greatly improves market efficiency and level of competition. Another way to increase the level of exploration is to directly and positively incentivize switching between new alternatives (Gneezy et al., 2020). For example, Shavit et al. (2021) considered unhealthy eating habits as a problem of insufficient exploration of healthy alternatives. These researchers found that incentivizing exploration of new fresh salads during a short intervention period increased (healthier) salad consumption in the long run. Thus, these examples suggest that to effectively tackle problems of insufficient exploration, one easy solution can be to develop interventions that reduce the direct costs (or increase the direct benefits) incurred when trying new options.

Yet, research also shows that the problem of insufficient exploration is not limited to preference for familiar (sub-optimal) options but can also occur at the strategy level (see also Mehlhorn et al., 2015). A ‘‘strategy’’ in this context can be defined as any plan, method or system for executing a set of choices or actions, implemented to obtain some subjectively valued goal^1^. Studies focusing on acquisition of skills demonstrate the benefits of training protocols that encourage exploration of new strategies (rather than new options). Seagull and Gopher (1994) demonstrated the value of such training schemes. By forcing more head movement during training in a flight simulator, participants greatly improved performance in a subsequent test, compared to a control group that could train without constraints. The researchers attributed the intervention’s success to its effectiveness in forcing participants to explore different strategies. Thus, forcing exploration of new options (e.g., new head movement) might sometimes be needed to encourage exploration of favorable strategies.

Note that in most cases, under-exploration of options coincides with under-exploration of strategies (as the examples above suggest). However, this is not always true. For example, an underlying strategy of avoiding long term commitment would likely correlate with going on many different dates. In this example, excessive exploration of alternatives (i.e., new dates) still implies reliance on the same underlying strategy (i.e., non-commitment). Thus, insufficient exploration of strategies can also manifest in over-exploration of options, leading to sub-optimal results. In such cases, reducing the costs of exploring new options (or even forcing exploration of new options) will only exacerbate the behavioral problem. Consider for example a person who eats on most weekdays at fast-food restaurants. This person might explore many fast-food items, alternating between alternatives and almost never repeating her choices. Yet, despite high exploration rates of new items, the underlying problematic behavior reflects a tendency to under-explore healthier eating strategies.

The examples highlighted above illustrate the importance of distinguishing between interventions that encourage exploration of options and interventions that encourage exploration of strategies. Unlike insufficient exploration of specific options, the problem of insufficient exploration of strategies might not be solved by direct reduction of costs (or increased prevalence of rewards) from exploring new strategies. This is because strategies (1) cannot be directly observed and (2) in many cases they are neither explicit nor conscious (i.e., trainees and consumers might not be aware of the specific strategy they use, e.g., see Dulany et al., 1984; Shanks and St. John, 1994; Newell and Shanks, 2014; Plonsky and Teodorescu, 2020). One indirect way to reduce the frequent costs associated with exploration of new strategies is to encourage experiences in which failures are not fully realized. For example, in competitive sports, exploring new techniques during a match is rare, as it will likely result in losing the competition. Yet practicing new moves during training can increase the chances of success in competitions as, in addition to perfecting one’s skills, it allows finding new ways to win. For example, the discovery of a great (but hard to find) set of moves that map particular responses to particular situations. Such a sequence of moves can be planned to anticipate opponent’s reactions and minimize the chance of a successful reply.

In the decisions from experience literature, many choice tasks allow participants to freely sample the outcomes of available options before committing to a consequential, binding choice. This might be taken as a similar situation to the examples above, as this experimental paradigm includes a clear “practice” stage. During this type of practice, the participants can freely explore the available outcomes of each alternative before executing their conclusive choice (Hertwig et al., 2004). Yet, the sampling paradigm does not allow to distinguish between exploration of options and exploration between sampling strategies. For example, high rates of alternation between two choice options might imply a participant’s attempt to either randomly explore the two options (e.g., Hills and Hertwig, 2010; Gonzalez and Dutt, 2016), or exploit a strategy that follows a specific pattern (see Cohen and Teodorescu, 2021; also see Schulze et al., 2020 for a similar observation in a probability matching task).

To examine the fundamental effect of practice (non-realization of payoffs) on exploration of strategies we aimed for a context-free task in which exploration is of particular importance. Our goal was to design a setting in which numerous possible strategies exist, and exploration of strategies can be distinguished from exploration of options. We focus on a repeated decisions-from-experience setting, in which participants do not receive *a priori* information about the payoff structure. This type of experimental tasks can be viewed as an example of an “ill-structured” problem (Simon, 1973; Wood, 1983; Reed, 2016). In a “well-structured” problem, the set stimuli (e.g., London subway system), the set of responses (e.g., possible routes from home to work) and the set of rules that determine their interaction are all clearly defined. Thus, such problems contain all the information needed to achieve the agent’s goal state. In contrast, in complex decisions-from-experience settings, participants must discover this information for themselves, requiring exploration of the task environment to be carried out over many of the trials.

In addition, to distinguish between exploration of options and exploration of strategies, the number of available choice options should be sufficiently large (i.e., correspond to the number of trials). This feature makes our settings very different than most common decisions-from-experience tasks. These latter tasks include the binary clicking paradigm (Barron and Erev, 2003), the sampling paradigm (Hertwig et al., 2004; Weber et al., 2004), the Iowa Gambling Task (Bechara et al., 1997), and probability learning tasks. These tasks typically present to participants only 2–4 options to choose from, and involve a relatively simple, static payoff rule (Cohen and Teodorescu, 2021). Accordingly, in these tasks exploration of new options is limited to the first few trials, precluding observations of behaviors that reflect under- and over- exploration of new alternatives. Similarly, to address exploration of strategies, our paradigm must allow for the execution of a sufficient number of strategies. Previous multi-alternative settings in decisions-from-experience studies (e.g., Teodorescu and Erev, 2014b) usually differentiate between only two meaningful strategies – exploration of new options and exploitation of familiar options. We extend the set of available strategies in our task by introducing a dynamic payoff rule (described below) that depends on the timing of exploration and the subject of exploitation. This ensures a large set of strategies that vary in their effectiveness, thus allowing for improvements at the strategy level. To achieve such improvements, one must first acquire costly information that can only be gained by exploration of the space. As the literature reviewed above suggests, reducing the cost of acquiring this information (by eliminating the costs of exploration) should increase the likelihood such a search will be effective.

Importantly, the above features were combined in a way that represents the elementary features of many real-life situations. Specifically, in the simplified task we developed, exploration of new options is frequently disappointing but can result in the discovery of great “treasure” options (henceforth referred to as “treasures”). Yet, finding one treasure is not enough, because repeated selection of the same treasure leads to depletion of the rewards it yields, while avoiding selection of the treasure replenishes its reward. Accordingly, to maximize returns in our task, one must develop a strategy that first finds several good options, and then alternate between them so that depletion of profit is minimized. For example, consider a person trying to integrate more green salads into her diet. Exploration of new types of salads is costly (purchasing the vegetables, peeling, cutting, etc.) and the result might not be very tasty compared to unhealthier but favored dishes. Given enough exploration efforts, one is likely to find a salad that is both healthy and very tasty. Yet eating this “treasure” salad every day will gradually reduce its appeal. Alternatively, if one will avoid eating this specific salad for a while, its attractiveness is likely to be renewed so that the salad becomes very tasty once again. In the context of competitive sports, it is very hard to find great moves that will defeat your opponent. After finding such a great move, using it repeatedly will likely make it predictable and reduce its value. Here too, avoiding this move for a while can restore its initial advantage. Thus, exploration between options in these examples can reflect choosing to eat a different salad every day or trying different moves against each different opponent. For the salad eater, exploration between strategies might reflect, for example, trying different rotation schedules between a handful favorite salads. In the context of competitive sports, it might reflect, for example, execution of one’s greatest moves only once against each opponent, at the most favorable time during the match.

In both examples, as in other situations reflecting similar incentive structures (i.e., rare treasures that deplete and replenish depending on recent usage), the optimal strategy involves a relatively complex combination of exploration and exploitation of options. For optimal results, one should explore new options until several “treasures” are discovered. After this initial exploration, the optimal strategy dictates alternating between the discovered treasures. Thus, an efficient exploration-exploitation policy is required to prevent value depletion and yield maximum results. To find this optimal strategy, one must first acquire some understanding of the underlying dynamics of the depleting and replenishing treasures. In our ill-structured task, this requires participants to apply flexible policies that involve exploration of the underlying rules and inhibition of unprofitable actions/hypotheses. Previous research in young children found the ability to solve such ill-structured problems (i.e., “tool-innovation” tasks) is difficult and late-developing (Cutting et al., 2011; Chappell et al., 2013). Thus, the complexity of the optimal strategy in the current multi-alternative setting implies that within the space of available strategies, the optimal strategy itself reflects a rare treasure. Accordingly, our setting implies a Rare Treasure environment at the strategy level.

We hypothesized that in the current setting (1) most participants will not find the optimal strategy due to insufficient exploration of different strategies. That is, participants will either not explore enough options to discover the good options and/or exploit treasures inefficiently (without avoiding depletion and/or without taking advantage of replenished values). (2) Practicing the payoff structure without realization of the payoffs will increase exploration of new strategies that are otherwise avoided due to the monetary losses associated with strategy exploration. More exploration between strategies is expected to enhance learning and lead to improved performance on a following task. In addition, previous research suggests that allowing participants to control the duration of practice can also improve learning and performance (e.g., Wulf et al., 2010; Wu and Magill, 2011). It was argued that self-controlled practice supports people’s need for autonomy, improves the level of (deeper) information processing while also increasing internal motivation (Lessa and Chiviacowsky, 2015). To examine whether self-controlled practice length helps people better explore the available strategy space, we also added a condition in which participants can terminate the practice trials whenever they wish.

## Study 1

### Methods

#### Participants

One-hundred and eighty-three participants (65 females, *M*_*age*_ = 30.3, *SD*_*age*_ = 9.5, and Range_*age*_ = [18, 62]) were recruited using Prolific Academic.^2^ Participants were informed they will earn a fixed show-up fee of 0.85£ (about 1.13$) and will also receive a bonus based on the outcome of their choice in one randomly selected trial. As bonus, participants received an endowment of 0.2£ (to offset potential losses) + the outcome of one randomly selected trial divided by six. Mean bonus was about 0.29£ (about 0.39$). The whole experimental session lasted 12 min on average.

#### Procedure

**FIGURE 1:**
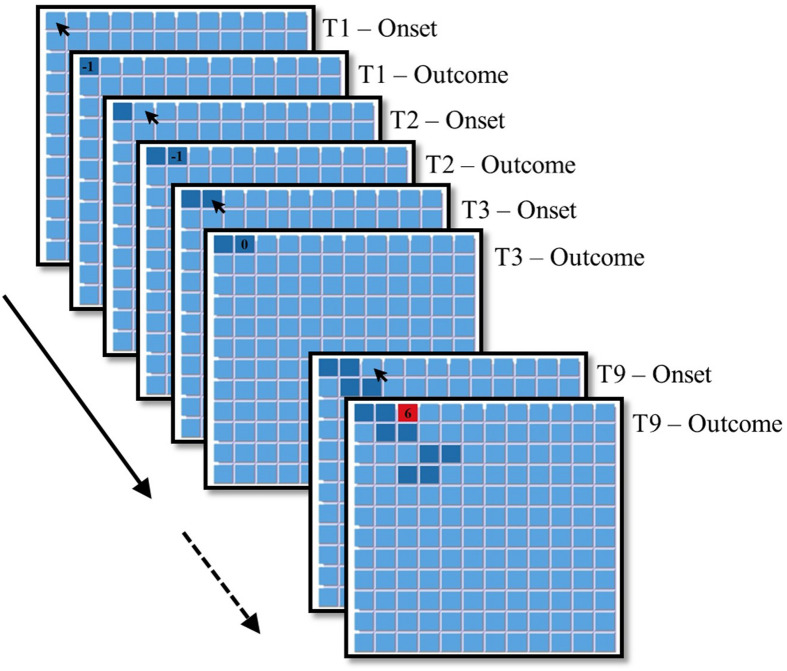
Examples of experimental screens for a hypothetical participant playing the first *T* = 9 trials in Study 1. Feedback regarding the trial’s outcome appeared for 1 s, after which the trial’s payoff disappeared and participants could choose again the same/another key.

The experiment consisted of two games. Each game presented a matrix of keys with an underlying payoff structure that represented a Rare Treasure environment (see Teodorescu and Erev, 2014a,b). A novel feature of the current task was that keys representing Treasures depleted and replenished as a function of their past exploitation rates. In each of the two games, participants faced a grid of 12 × 12 unmarked keys (see Figure 1). Participants were told that the first game they will face includes 75 trials and the second includes 150 trials (the matrix was reset after the first game; see full instructions in the online Supplementary Appendix^3^). Importantly, participants were not informed about the underlying payoff structure of the task. The participants were informed that their task in each trial is to choose one of the keys, and that each choice could either give them points or cause them to lose points. Immediately after each choice the trial’s payoff appeared on the selected key for 1 s. Afterward, the feedback disappeared, and the participants could choose again. Immediately after each key was first selected, its color changed to either dark blue (for Non-treasure keys) or red (for Treasure keys; see Figure 1).

The underlying payoff structure was a modified version of the Rare Treasure game studied in Teodorescu and Erev (2014a, b). Exploration of a new key (that was not selected in previous trials in that game) either led to a small loss with probability 0.9 (i.e., a Non-treasure), or a large gain (i.e., a Treasure) otherwise. A Non-treasure key always implied a loss of 1 point the first time it was chosen and yielded an outcome of 0 on any subsequent selection. A Treasure key yielded on first selection a payoff of six points. The value of the same Treasure key on subsequent trials depended on the number of times that key was selected in the last *k* trials. Table 1 presents the payoff rule we used in the current experiment. This rule implies that the value of a Treasure key was equal to 2^∗^(*k* -- *n*[*T*(*i*)]), where *n*[*T*(*i*)] equals the number of times Treasure *T*(*i*) was selected on the last *k* trials. In the current experiment, *k* was set to equal 3.^4^

**TABLE 1:** The effect of previous choices on the value of treasure keys with *k* = 3.

Example	*n*[*T(i)*] before the current trial	*T(i)* payoff value in the current trial
*T(i)* key was not selected in the prior 3 trials	0	6
*T(i)* was selected only once in the prior 3 trials	1	4
*T(i)* was selected twice in the prior 3 trials	2	2
*T(i)* was selected three times in the prior 3 trials	3	0

Participants were randomly allocated to one of three conditions. In Condition “No Practice,” participants were informed that out of the total 225 trials (75 trials in Game 1, 150 in Game 2), one trial will be chosen at random, and that trial will determine their final payoff. In Condition “Forced Practice,” participants were told the first game is dedicated to practicing the task and will not influence their final payoff. They were told only choices in the second game will influence their earnings (i.e., one trial out of the 150 trials of Game 2 will be selected randomly to determine the final payoff). In Condition “Free Practice,” participants received the same instructions but were also provided with an option to stop practice and start the “real” game at any point during the 75 trials of Game 1. They were told that once they indicate they wish to stop the practice stage, any following trials in the first (and second) game can also determine their final payoff (nothing else changed). See full instructions in the online Supplementary Appendix. We had 63, 60, and 60 participants in Condition No Practice, Forced Practice and Free Practice, respectively.

#### Analysis of Different Strategies Using Computer Simulations

The top five rows of Table 2 present the expected outcomes of different strategies implying different levels of sophistication, using computer simulations of the above task. Although an infinite number of possible choice strategies exist for the current task, we chose to simulate these specific five strategies because they represent two extremes of the possible strategy space. While the top three strategies represent the optimal and local-maxima extremes, the bottom two strategies represent an extreme of naivete, characterized by random choice.

**TABLE 2:** Descriptive results of Game 2 in simulations and Conditions observed in Studies 1 and 2.

	Outcome per trial	Exploration rates	# Treasures found	# Exploitation of treasures	# Total keys exposed
	
	***Mean, Game 2 [95% CI]***				
**Strategy simulations**					
Find *T* = 4, then rotate (exploit)	4.3	0.27	4	114	38.9
Find *T* = 3, then rotate (exploit)	3.2	0.20	3	123	28.8
Find *T* = 2, then rotate (exploit)	3.44	0.13	2	132	18.7
Random, with replacement	0.04	0.62	9.3	15	89.3
Random, without replacement	−0.24	0.96	14.4	16	138.2
**Study 1**					
No practice	0.42	0.81	12.6	34.7	119.1
	[0.1, 0.72]	[0.75,0.87]	[11.3, 13.8]	[26, 43]	[110.6, 127.7]
Forced practice	0.53	0.83	11.9	36.7	116.6
	[0.2, 0.85]	[0.77,0.89]	[10.5, 13.2]	[28, 45]	[107.4, 125.7]
Free practice	0.26	0.85	13.2	30.4	121.7
	[0.02, 0.5]	[0.79,0.90]	[12.1, 14.4]	[23, 38]	[113.6, 129.7]
**Study 2**					
Forced practice, constrained	1.04	0.73	10.7	48.0	105.1
	[0.62, 1.46]	[0.65,0.80]	[9.5, 11.9]	[38, 58]	[93.6, 115.2]
Free practice, constrained	1.16	0.72	11.4	52.2	103.7
	[0.77, 1.55]	[0.64,0.79]	[9.9, 12.8]	[42, 63]	[92.2, 113.8]

*T refers to treasure keys. Random strategy implies one key randomly selected in each trial. Exploration rates refer to % of choosing new (unexploited) keys in the 150 trials of Game 2. #Treasures found and #Exploitation of Treasures refer to the number of Treasure keys discovered and the number of trials these were chosen, over the 150 trials of Game 2, respectively. #Keys exposed refers to the average number of keys explored (i.e., uncovered) over the 150 trials of Game 2 (out of the possible 144 keys).*

The top row presents the optimal strategy: it assumes exploration of new keys until four Treasures have been found, and then assumes a strict policy of rotation between these Treasures. This policy relies on the replenishing nature of Treasures to ensure each selection maximizes the treasure’s value. The next two rows present “local maxima” strategies, i.e., rotation between three or two Treasures. The following rows present the expected outcomes of executing random choice strategies. We consider two types of “random” strategies. In the first, on each trial, the agent draws one key with replacement from the 144 possible keys (i.e., repetition is possible) and chooses that key. In the second type of “random” strategy, on each trial, the agent draws one key without replacement (i.e., no repetition) from the remaining (144 – #trial) keys and chooses that key on that trial. In the final 6 trials, the agent draws without replacement from the 144 keys again (i.e., the sample is reset).

### Results

Analysis of Game 1 (75 trials) shows no difference in either mean outcome or exploration rates (of new keys) across the 3 conditions (see Supplementary Figure A1 in the online Supplementary Appendix for a detailed presentation of Game 1’s result). The mean outcome per trial in Game 1 was −0.06 (95% CI [−0.24, 0.12]), −0.07 (95% CI [−0.23, 0.09]), and −0.01 (95% CI [−0.18, 0.15]) for Condition No Practice, Forced Practice and Free practice, respectively. In Condition Free Practice, the practice phase was stopped on average after 35.7 trials, 95% CI [28.8, 42.6]. That is, participants stopped practice after about 48% of the possible number of practice trials. We calculated the Spearman rho correlation coefficient to evaluate the relationship between the number of practice trials at Game 1 and mean outcome per trial in Game 2. This correlation was not significant, *r*_*S*_ = 0.033, 95% BCa CI [−0.220,0.292], *p* = 0.802, *N* = 60.

**FIGURE 2:**
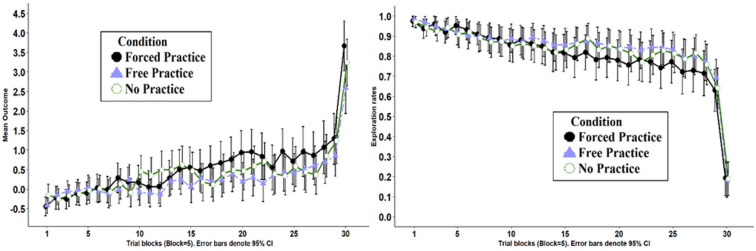
Left: mean outcome in trial blocks in Game 2 (each block equals five trials), across the three Conditions of Study 1. Right: choice rates implying choice of unexplored keys in Game 2 (each block equals five trials), across the three Conditions of Study 1. The right panel of Figure 2 presents the mean exploration rates across the 150 trials in the three Conditions.

Table 2’s middle rows present the main results of Game 2, which was equivalent across all three Conditions. The left panel of Figure 2 presents the mean outcome across the 150 trials in Game 2, aggregated to blocks of five trials, for the three Conditions. To test for statistical significance we used a linear mixed-effects model (using R packages lme4, Bates et al., 2015; and lmerTest, Kuznetsova et al., 2017). We set random intercepts for participants, and fixed effects for Condition (three levels: No Practice, Forced Practice, and Free Practice) and Trial number (1–150, treated as a continuous variable) along with their interaction. The main effect for Condition (χ^2^(2) = 1.81, *p* = 0.404) was not statistically significant. We find a significant main effect for Trial (χ^2^(1) = 1042.0, *p* < 0.001), suggesting mean outcome increased significantly over trials in all the conditions. The interaction between Condition and Trial was also statistically significant (χ^2^(2) = 41.11, *p* < 0.001). A *post hoc* analysis shows the outcome increased as a function of trial in all three Conditions. This increase was more pronounced in Condition Forced Practice, *b* = 0.014, 95% CI [0.012,0.015], *t*(26911) = 22.5, and *p* < 0.001, compared with the No Practice Condition, *b* = 0.010, 95% CI [0.009,0.011], *t*(26911) = 17.7, and *p* < 0.001, and Free Practice Condition, *b* = 0.009, 95% CI [0.007,0.098], *t*(26911) = 15.1, and *p* < 0.001.

The right panel of Figure 2 presents the mean exploration rates of the options across the 150 trials in Game 2, aggregated to blocks of 5 trials, for the 3 Conditions. We define exploration as choice of an unfamiliar key (i.e., that was not selected in previous trials). A logistic mixed-effects model^5^ shows the main effect for Condition (χ^2^(2) = 0.89, *p* = 0.64) was not statistically significant. We find a significant main effect for Trial (χ^2^(1) = 3184, *p* < 0.001), and a significant interaction between Trial and Condition (χ^2^(2) = 27.56, *p* < 0.001). *Post hoc* tests reveal the same pattern of results as the analysis of mean Outcome presented above.

### Discussion

The results of Study 1 suggest participants in all three conditions did not find and exploit any of the maximizing strategies (see Table 2). Rather, most participants seemed to rely heavily on a strategy that implies over-exploration of the choice alternatives. Thus, participant’s tendency to over-explore alternatives implies under-exploration of choice strategies. Although allowing participants to explore between different strategies with no costs (i.e., in the Free and Forced Practice Conditions) did not alleviate this tendency, it did lead to somewhat improved learning rates in the Forced practice Condition. Conversely, in the Free Practice Condition, in which participants were free to decide when they had trained enough, learning rates and average score in Game 2 were the lowest (see Table 2). Participants in this condition exhibited a stronger tendency to over-explore alternatives, and thus had worse outcomes than in the other two conditions. This result is inconsistent with our initial hypothesis that removing the costs entailed by exploring among strategies will increase exploration of strategies and improve performance.

The results of Study 1 suggest that removing the costs associated with exploration *via* an explicit practice phase (in which payoffs are not realized) is not enough to encourage exploration between choice strategies. One possible reason is that non-realization of payoffs reduces only the exogenous costs of exploration. However, exploration of new strategies might also involve endogenous costs, e.g., requiring investment of additional cognitive/mental resources. This likely increases the perceived effort of exploring new strategies, highlighting opportunity costs of investing one’s mental resources in potentially more attractive non-task activities (such as daydreaming; Kurzban et al., 2013). Moreover, it is possible that many participants in our ill-structured task might be oblivious to the existence of many of the possible strategies (Sobolev et al., 2016), potentially increasing further the perceived opportunity costs. For example, it is possible that many participants explored only relatively easy strategies (e.g., always explore new keys; find a treasure and exploit it until depletion) because they did not realize that more complex strategies could be much more rewarding (outweighing the perceived opportunity costs).

Therefore, one potential solution is to reduce the perceived opportunity costs of trying more complex strategies. To this aim, in Study 2 we impose a constrained practice intervention which ensures that participants can experience the potential value of different strategy components. This approach is in line with Yechiam et al. (2001), which imposed constraints that encourage participants to try alternative strategies. Specifically, Yechiam et al. (2001) observed that preventing repetition of familiar options in some of the trials led to improved performance. Yet while the behavior in their experimental task revealed a tendency to under-explore the available options, in our (ill-structured) task the natural inclination seems to reflect over-exploration of options. Thus, instead of enhancing exploration of new options (as in Yechiam et al., 2001), our intervention encourages participants to *exploit* familiar options during some of the practice trials (rather than allowing unconstrained exploration). We hypothesized that this simple intervention during practice would lead participants to learn that better strategies exist. If indeed the main obstacle is under-exploration of strategies, positive experiences with strategies that involve exploitation of options might reveal the value of different (and perhaps less intuitive) policies. Such positive experiences could in turn outweigh the opportunity costs associated with strategy exploration and thus improve performance during the test phase (i.e., during Game 2). Study 2 tests this hypothesis.

## Study 2

### Methods

#### Participants

One-hundred and twenty-two participants (51 females, *M*_*age*_ = 30.0, *SD*_*age*_ = 10.5, and Range_*age*_ = [18, 63]) who explicitly did not participate in Study 1 were recruited using Prolific Academic (see text footnote 1). Participants were informed they will earn a fixed show-up fee of 0.85£ (about 1.13$) and will also receive a bonus based on the outcome of their choice in one randomly selected trial. In Study 2, as the bonus, participants received an endowment of 0.1£ + the outcome of one randomly selected trial with a conversion rate of one point = 0.05£. Mean bonus was about 0.17£ (about 0.22$). The whole experimental session lasted 11.46 min on average.^6^ We had 61 participants in each of the two conditions.

#### Procedure

The two conditions in Study 2 were identical in design and instructions to conditions Forced Practice and Free Practice (from Study 1), with the following addition: during the practice phase in Game 1, participants were required in a portion of the trials to only choose keys they already pressed in previous trials (i.e., to exploit rather than explore keys). Specifically, participants could choose freely for the first 10 trials of Game 1, after which they were constrained to choose only familiar keys for five consecutive trials^7^. After these five “constrained” trials, participants could again choose freely for the next 10 trials. This pattern repeated until the end of Game 1 (in the Forced Constrained Practice Condition) or until participants indicated they wish to stop practice (in the Free Constrained Practice Condition). Thus, in a full 75 trial practice phase participants experienced five constrained blocks of five trials each, each immediately following a 10-trial period of free choice. In the Forced Constrained Practice Condition, participants had to play Game 1 under this rule for 75 trials. In the Free Constrained Practice Condition, participants could choose to stop practice at any point during the 75 trials. Once they indicate this, the constraints were removed, and any subsequent choices could be realized.

### Results

Analysis of Game 1 shows a large impact of the manipulation. Overall, mean outcome in Game 1 was 0.94 (95% CI [0.71, 1.16]) and 0.56 (95% CI [0.38, 0.80]) in Condition Forced Constrained Practice and Free Constrained Practice, respectively (see Supplementary Figure A2 in the online Supplementary Appendix for a detailed presentation of this result). In Condition Free Constrained Practice, the practice phase was stopped on average after 40.2 trials, 95% CI [32.8, 47.5]. That is, participants stopped practice after about 54% of the possible number of practice trials. We also calculated the Spearman rho correlation coefficient to evaluate the relationship between the number of practice trials at Game 1 and mean outcome per trial in Game 2. Unlike the results of the Free Practice Condition in Study 1, this correlation for the Free Constrained Practice Condition was significant, *r*_*S*_ = 0.291, 95% BCa CI [0.043,0.512], *p* = 0.023, and *N* = 61. That is, a longer practice phase in Game 1 was related to improved performance in Game 2, providing additional support for the effectiveness of the constrained practice.

**FIGURE 3:**
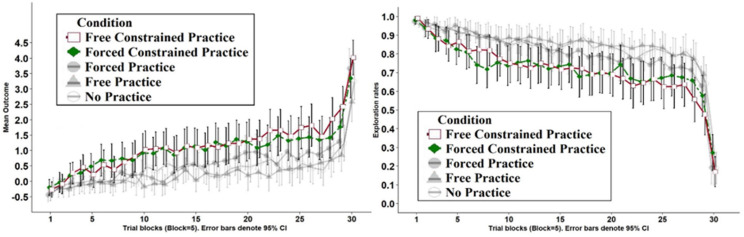
Left: mean outcome in trial blocks in Game 2 (each block equals five trials), across the two Conditions of Study 2. Right: choice rates implying choice of unexplored keys in Game 2 (each block equals five trials), across the two Conditions of Study 2. Shaded lines present the three Conditions of Study 1 for comparison.

The current analysis focuses on Game 2, which was equivalent across all conditions. Table 2’s bottom rows present the main results of this task, for the two “constrained” Conditions analyzed in Study 2 (this task was the same across all studies and conditions). The left panel of Figure 3 presents the mean outcome across the 150 trials in Game 2, aggregated to blocks of five trials, for these two “constrained” Conditions (it also compares the results in Study 1). First, we analyze the two constrained Conditions using the same statistical analysis as reported for Study 1. We find that the main effect of Condition was not statistically significant (χ^2^(1) = 0.18, *p* = 0.672). The main effect for Trial was significant (χ^2^(1) = 1118.3, *p* < 0.001). The interaction between Condition and Trial was also statistically significant (χ^2^(1) = 21.78, *p* < 0.001). A *post hoc* analysis shows that the increase in outcome implied a higher slope in Condition Free Constrained Practice, *b* = 0.017, 95% CI [0.015,0.018], *t*(18156) = 27.3, *p* < 0.001 compared to the Forced Constrained Practice Condition, *b* = 0.013, 95% CI [0.011,0.014], *t*(18156) = 20.78, *p* < 0.001.

To test the significance of the manipulation, we compare the two constrained Conditions and its corresponding two non-constrained practice Conditions (from Study 1) on per-trial outcome in Game 2. We use a linear mixed-effects model with two dummy variables: Practice Type (2 levels: Forced/Free) and Constraint (2 levels: With-Constraint/No-Constraint) and Trial number (1–150, treated as a continuous variable), along with the two and three-way interactions. We find a statistically significant three-way interaction, (χ^2^(1) = 57.48, *p* < 0.001). Considering the simple slopes between each Condition and Trial (described above), this three-way interaction suggests that a Free Practice manipulation implies better results when coupled with Constrained practice (compared to the Forced Practice Conditions). The same implies worse results when coupled with Non-constrained practice, compared to the Forced Practice Conditions. The two-way interaction between Constraint and Trial was also statistically significant, (χ^2^(1) = 34.69, *p* < 0.001). This implies that overall, learning rates were higher in the Constrained Conditions. Conversely, the two-way interactions between Practice Type and Trial, (χ^2^(1) = 0.56, *p* = 0.455), and Constraint and Practice Type, (χ^2^(1) = 1.30, *p* = 0.254), were not statistically significant. We found a significant main effect for Trial, (χ^2^(1) = 1844.80, *p* < 0.001) and for Constraint, (χ^2^(1) = 15.77, *p* < 0.001). The main effect of Practice Type was not statistically significant, (χ^2^(1) = 0.20, *p* = 0.660). Overall, analysis of the two-way interactions and main effects suggest that the main driver in the improvement of outcomes is the Constraint manipulation. Supplementary Figure A3 (in the online Supplementary Appendix) presents the average per-trial outcome for individual participants in each condition of Studies 1 and 2.

### Discussion

The results of Study 2 suggest that forcing repeated exploitation of options during practice can reduce the tendency to under-explore different strategies in our ill-structured task. Furthermore, our results suggest that a manipulation focused on constraining choices in a subset of practice trials can significantly improve performance in a subsequent task. This was especially evident when comparing performance in the Free Practice Conditions: while allowing participants to decide when to stop practice led to the worst outcomes in Study 1, combining this option with constrained exploration led to the best overall performance. Overall, the mean outcome per trial was almost twice as large in the Constrained Practice conditions (compared to the non-constrained conditions).

## General Discussion

The current work demonstrates the importance of distinguishing between behaviors that imply suboptimal exploration of options and behaviors that imply suboptimal exploration of strategies. While the former has been shown to be the root of many real-life problems (e.g., inertia biases), the current work is the first to investigate the distinct importance of the latter. Participants in our study faced two ill-structured exploration games with rare treasures that depleted following successive exploitation and replenished after a short period of abstinence. We manipulated the type of practice participants underwent during the first, shorter game and compared their performance in a second, longer game with an identical payoff structure.

While participants in the current task could employ an infinite number of strategies, our result show participants overwhelmingly preferred strategies that implied over-exploration of options. One explanation for this empirical result suggests our participants were strongly biased toward under-exploration of the space of available strategies. This explanation suggests that adverse behaviors, which can imply both under- and over-exploration of options, are in fact manifestations of under-exploration between choice strategies. In such cases, interventions that aim to improve the way people explore between options (e.g., by reducing the direct costs of exploring new options and/or by increasing the direct benefits of trying new options) might not increase, and potentially even reduce, exploration between strategies. Our result show that this type of oversight can reduce the chance that interventions have a beneficial, lasting effect.

The current results also suggest a boundary condition for the assumption of a general exploratory mode (Hills et al., 2008, 2015). Specifically, a general exploratory mode assumes a co-dependence between internal search (e.g., search for a better strategy or plan) and external search (e.g., search between options) processes. Our results clarify this assumption, showing that these two modes can be disentangled in environments where a tendency to over-explore the external space implies a tendency to under-explore the internal space. Future research can further investigate this distinction, clarifying the environments in which external and internal search coincide (see Hills et al., 2008, 2012, 2015).

We manipulated the amount and type of practice participants experienced before playing the longer, second game. In Study 1, we had three groups, a No Practice group, a group that was forced to practice throughout the entire first shorter game (Forced Practice), and a group that could stop practice whenever they felt ready (Free Practice). Although the task presented an infinite number of strategies (i.e., an infinite problem space, see Reed, 2016), there were only a few value maximizing strategies within the strategy space. These optimal strategies involved a relatively complex balance between exploration of unfamiliar keys and exploitation of uncovered treasures, and a policy of rotation between treasure keys when enough have been found.

As this optimal solution is not intuitive or easy to find, we hypothesized that a practice period (in which exploration of new strategies do not bear monetary losses) would encourage exploration of different solution strategies. This, we expected, would increase the likelihood that participants learn an outcome-maximizing choice strategy. The results suggest otherwise, as participants preferred to persist with a strategy that implies over-exploration of the available options. Therefore, it seems that simply removing the monetary costs associated with exploration of strategies is not sufficient to improve performance. One likely explanation relies on the assumption that, much like in real life, exploration of new strategies entails substantial endogenous costs (e.g., cognitive/mental effort) on top of the exogenous costs (i.e., monetary losses). That is, while non-realization of payoffs during practice removes exogenous costs of exploration, it does not eliminate endogenous mental costs. Thus, it could be that participants did not sufficiently explore the set of possible strategies due to perceived effort and/or high opportunity costs (Kurzban et al., 2013).

To reduce the overall opportunity costs associated with exploration of new strategies, in Study 2 we aimed to expose participants to the benefits of less intuitive strategies. Specifically, we forced participants to exploit familiar options in some of the practice trials. We hypothesized that such a manipulation would expose participants to the potential of more complex strategies that integrate exploration and exploitation of options (rather than simpler classes of strategies, e.g., always-explore-new-options). The results show that the constrained practice manipulation indeed improved later performance, almost doubling the mean outcome per trial in Study 2 (compared with Study 1). Moreover, in the free-practice condition without constraints (Study 1), we found no significant correlation between practice length and subsequent performance. In contrast, in the constrained free-practice condition of Study 2, a longer practice period was significantly associated with improved performance, providing an additional support for the effectiveness of the constrained practice. Taken together, these results demonstrate the potential of exposing trainees to the benefits of different strategy components *via* imposition of constraints during practice. This conclusion is in line with a study by Finke (1990) that focused on creativity in a product design task. This study found that putting multiple constraints on participant’s designs improved both originality and novelty of their products. The conclusion was similar to ours – that the constraints impeded reliance on familiar (but sub-optimal) solutions to the problem (Finke, 1990).

Comparison of the effects of the forced- vs. free- practice duration revealed an interesting interaction. On average, participants in the Free Practice groups in both studies went through about 50% of the practice trials experienced in the Forced Practice Conditions. Yet, while the Free Practice group exhibited the worst performance in Study 1, combining free practice with constrains on exploration (i.e., during practice) in Study 2 led to the best performance overall. This finding can be especially important as many types of real-world interventions are very costly. Thus, understanding the conditions that allow maximizing their impact while minimizing their duration can be extremely valuable. Our results suggest that experiencing the outcomes of different strategies during practice is key to control the efficacy of shorter interventions. The current study can also shed light on the efficacy of “Emphasis Change” training protocols (Gopher et al., 1989, 2007; Gopher, 1993). This method focuses on repeated changes to the perceived priorities, from the perspective of trainees, of different elements during training of a complex task. Previous studies have shown this method improved performance significantly, compared to more complex training schemes (Yechiam et al., 2001; Gopher et al., 2007; Lee et al., 2012). Yechiam et al. (2001) suggested that the Emphasis Change protocol is primarily successful because of its exploration-enhancing potential (see also Erev and Gopher, 1999). That is, it encourages exploration of options in settings in which people do not explore enough. Our study clarifies the type of exploration that is crucial for the success of such training schemes in ill-structured problems. Emphasis Change is expected to lead to significant long-term learning and skill transfer primarily when it encourages exploration among strategies rather than among options (see Lee et al., 2012 for related results).

Another way to learn about the possible payoffs without realizing the costs of exploration is through social observations of the consequences of other people’s choices (e.g., Yechiam et al., 2008). The fact that in our study non-constrained practice did not help people find better solutions is in line with studies focused on this type of “social exposure” to other’s outcomes. For example, Yechiam et al. (2008) found that observing other’s outcomes led participants to exhibit more underweighting of rare events. Our findings suggest that social learning can be improved if decision makers are exposed to other players that explore the strategy space rather than explore different options.

Our results also demonstrate that in rare treasures settings, the frequently observed tendency to under-explore is robust at the strategy level, but not at the individual choice level. That is, when the optimal strategy is complex and reflects a rare treasure, people seem to under-explore the space of available strategies. In such settings, people’s policies of choice might converge to a narrow set of sub-optimal strategies that can also manifest as over-exploration of options. Accordingly, trainers and guides should focus on encouraging exploration of new strategies, rather than encouraging exploration of new options. In fact, in our task, encouraging exploration of new options directly, for example by rewarding participants for trying new options (e.g., with a bonus payoff, see Shavit et al., 2021), would be counterproductive as it would have increased the usage of suboptimal strategies.

On another practical level, our results demonstrate the potential of interventions that force exploitation of options to improve real-life outcomes and satisfaction. One example relates to online dating apps (Tinder is one example). Online apps are purported to help reduce the costs of exploration of dating opportunities, and by doing so increase the chance its users establish meaningful relationships (Finkel et al., 2012), which in turn have been shown to increase long-term life satisfaction (see Lehmann et al., 2015). Yet, our results suggest that reducing the costs of exploration of options might not help increase exploration of strategies. Instead, our results suggest users might prefer to over-exploit a specific strategy that can imply over exploration of dating opportunities, thus reducing the chances of finding meaningful partnership (e.g., Rochat et al., 2019). The current work also suggests that a short intervention that encourages exploitation of familiar options (or limiting the chance to explore new options) can improve subsequent outcomes for users, by showing that better strategies exist.

Future studies are needed to validate the practical implication of our results in different real-life contexts. For example, it is possible that in highly engaging environments, curiosity naturally increases exploration of strategies, making constrained practice redundant and even potentially harmful. In addition, it is important to note that the focus of the current work is on an ill-structured experimental design, in which the information needed to achieve one’s goal is not readily available. This lack of information necessitates exploration by trial-and-error of different strategies and options, mimicking natural experience-based activities such as dating, dietary decisions, and practicing competitive sports. In contrast, well-structured tasks can convey much more information (e.g., a description of the payoff structure), greatly reducing the costs of exploration between different solution strategies. Future studies can test whether the effect of under-exploration of strategies also holds when such information is readily available. Lastly, an important open question is whether and how individual’s personality characteristics mediate the relationships between practice, exploration of options, exploration of strategies, and performance. For example, the positive relationship between practice length (in the free-constrained practice condition of Study 2) and performance on the subsequent task (i.e., Game 2) might be driven by individual risk-taking and/or curiosity tendencies (e.g., people with lower need for cognition and/or higher risk tendencies could practice for shorter periods and also explore new options more). Therefore, a better understanding is needed of how disposition toward risk taking and need for cognition interact with exploration between options and strategies. For example, such understanding can contribute to the development of effective tailored training protocols (e.g., see Schaefer et al., 2010).

## Data Availability Statement

The datasets presented in this study can be found in online repositories. The names of the repository/repositories and accession number(s) can be found below: https://osf.io/m6gdz/.

## Ethics Statement

The studies involving human participants were reviewed and approved by the Social and Behavioral Sciences Institutional Review Board (IRB), Technion. The patients/participants provided their written informed consent to participate in this study.

## Author Contributions

DC and KT came up with the idea and designed the experiment. DC programed the experiment with feedback from KT. DC collected and analyzed the data. Both authors contributed to the article and approved the submitted version.

## Conflict of Interest

The authors declare that the research was conducted in the absence of any commercial or financial relationships that could be construed as a potential conflict of interest.

## Publisher’s Note

All claims expressed in this article are solely those of the authors and do not necessarily represent those of their affiliated organizations, or those of the publisher, the editors and the reviewers. Any product that may be evaluated in this article, or claim that may be made by its manufacturer, is not guaranteed or endorsed by the publisher.

## Footnotes

1 We define the “goal” in this context very broadly. This can be a conscious goal set in advance by the decision maker (e.g., maximize profit from each individual choice), or unconscious (invest as little effort as possible in the task). We assume each goal is situation specific and flexible regarding the strategy it implies.

2
https://prolific.ac

3
https://osf.io/m6gdz

4 For example, assuming *k* = 3 (the task parameter k is defined *a priori*), in the first selection of a *T(i)* key, *n*[*T(i)*] = 0, i.e., the number of times this key was selected in the last three trials is zero. In such a case, the Treasure *T(i)* value will be six. If the same *T(i)* key is selected again on the subsequent trial, *n*[*T(i)*] = 1 and the Treasure *T(i)*’s value will be four. On the 3rd consecutive selection of the same *T(i)* key, *n*[*T(i)*] = 2, and *T(i)*’s value will be two. With further consecutive selections of this key, *n*[*T(i)*] = 3 and *T(i)*’s value will be zero. However, if after discovering a Treasure *T(i)*, one presses other keys for at least three consecutive trials, *n*[*T(i)*] will be reset and Treasure *T(i)* value will be six again.

5 As Laplace approximation did not converge (although yielding nearly identical results), we used the penalized least squares method for parameter estimation.

6 Due to a technical mistake, Study 2 used a slightly different payoff rule than Study 1. This mistake implied that in Study 2 participants received a smaller positive payment from choosing treasure keys. Yet, our results (see below) suggest that if anything, the manipulation should be even stronger with an equivalent payoff rule to that used in Study 1.

7 Although five constrained trials (on which participants are forced to explore familiar keys) represent a somewhat arbitrary number, we chose it as it was similar to the manipulation of forced exploration in Yechiam et al. (2001) Experiment 1. In Yechiam et al.’s experiment, participants were forced to only explore unfamiliar option for five consecutive trials during their practice phase.
