# Supplementary material for: On the Effect of Practice on Exploration and Exploitation of Options and Strategies
Source: Front Psychol. 2021 Nov 12;12:725690. doi: 10.3389/fpsyg.2021.725690 (PMC8632697; doi:10.3389/fpsyg.2021.725690)
Supplement: Supplementary file 1 [file Data_Sheet_1.docx]

Supplementary Material

Contents

[1 Supplementary Appendix A 1](#_Toc84885395)

[1.1 Instructions in Study 1. 1](#_Toc84885396)

[1.1.1 Instructions for participants in Condition “No Practice” 1](#_Toc84885397)

[1.1.2 Instructions for participants in Condition “Forced Practice” 1](#_Toc84885398)

[1.1.3 Instructions for participants in Condition “Free Practice” 2](#_Toc84885399)

[1.2 Instructions in Study 2. 2](#_Toc84885400)

[1.2.1 Instructions for participants in Constrained Practice Conditions 2](#_Toc84885401)

[2 Supplementary Appendix B: Results in Game 1 3](#_Toc84885402)

[3 Supplementary Appendix C: Individuals performance in both studies 4](#_Toc84885403)

# Supplementary Appendix A

## Instructions in Study 1.

### Instructions for participants in Condition “No Practice”

*"The current experiment consists of 2 decision-making games. The main game includes 150 trials. Before starting the main game, you will face a shorter game for 75 trials. In each game you will face a grid of squares, and in each trial your task is to choose one of the squares. Sometimes choosing squares will give you points, and sometimes choosing the squares will cause you to lose points.*

*Your bonus payment will be determined by the outcome of one randomly selected trial from the experiment. These can be any of the 75 + 150 trials you will face. Note - the more trials with a higher outcome, the better your chance to receive a higher payment at the end of this experiment.*

*Your bonus payment will be the outcome of one randomly selected trial from the experiment, divided by 6. These can be any of the 75 + 150 trials you will face. Your payoff from the current experiment will comprise of a fixed amount for participation of 0.85£, promised upon completion. Beside this amount, you will be paid a bonus comprised of an endowment of 0.2£ + your outcome in one randomly selected trial from the two games (if positive it would be added to the endowment and if negative it would be subtracted from the endowment), divided by 6. So, for example, if in the randomly selected trial you earned 2 points, your final payoff will be 2/6 = 0.33£ + 0.2£ = 0.53£. This will be added to the promised payment of 0.85£. The final payoff will be presented at the end of the experiment."*

### Instructions for participants in Condition “Forced Practice”

*"The current experiment consists of a game of 150 trials. Before starting the final game, you will face a shorter game for 75 trials. In each game you will face a grid of squares, and in each trial your task is to choose one of the squares. Sometimes choosing squares will give you points, and sometimes choosing the squares will cause you to lose points. In the first game you will face, you will be able to practice the task. Once you finish the 75 practice trials, you will automatically start the 'real' experiment, and play the task again for 150 trials.*

*Your bonus payment will be determined by the outcome of one randomly selected trial from the 'real' experiment. Once you finish the full practice game (75 trials), you will face the same game for 150 trials, and will be paid the outcome of one randomly selected trial of the 150 trials you played 'for real'.*

*Your payoff from the current experiment will comprise of a fixed amount for participation of 0.85£, promised upon completion. Beside this amount, you will be paid a bonus comprised of an endowment of 0.2£ + your outcome in one randomly selected trial from the second game (if positive it would be added to the endowment and if negative it would be subtracted from the endowment), divided by 6. So, for example, if in the randomly selected trial you earned 2 points, your final payoff will be 2/6 = 0.33£ + 0.2£ = 0.53£. This will be added to the promised payment of 0.85£. The final payoff will be presented at the end of the experiment."*

### Instructions for participants in Condition “Free Practice”

*"The current experiment consists of a game of 150 trials. Before starting the final game, you will face a shorter game for 75 trials. In each game you will face a grid of squares, and in each trial your task is to choose one of the squares. Sometimes choosing squares will give you points, and sometimes choosing the squares will cause you to lose points. In the first game you will face, you will be able to practice the task as much as you need. But, although you can have the whole 75 trials for practice, you can also decide to advance and start playing the experiment before the 75 trials have passed. You will be paid for only one of the none-practice trials, so once you decide to stop practicing (or otherwise you finished the 75 trials) the 'real' game begins. Once you feel you are ready, press the yellow button at the bottom of the screen. Once you press the button (or the first game has ended), You will start the actual experiment.*

*Your bonus payment will be determined by the outcome of one randomly selected trial from the 'real' experiment. So, for example, if you decided to play the full practice game (75 trials), you will face the second game for 150 trials, and will be paid the outcome of one randomly selected trial of the 150 trials you played. If, say, you decided to stop practicing after 3 trials, your bonus payment will be the outcome of one randomly selected trial from the 150 + 72 = 222 trials you played 'for real'.*

*Your payoff from the current experiment will comprise of a fixed amount for participation of 0.85£, promised upon completion. Beside this amount, you will be paid a bonus comprised of an endowment of 0.2£ + your outcome in one randomly selected trial from the trials you played 'for real' (if positive it would be added to the endowment and if negative it would be subtracted from the endowment), divided by 6. So, for example, if in the randomly selected trial you earned 2 points, your final payoff will be 2/6 = 0.33£ + 0.2£ = 0.53£. This will be added to the promised payment of 0.85£. The final payoff will be presented at the end of the experiment."*

## Instructions in Study 2.

### Instructions for participants in Condition “Forced Practice Constrained” and “Free Practice Constrained”

See respective instructions for each Condition in Study 1 (as presented above), with the following addition:

“*During practice mode (only on the first game), new rules can be introduced. You will be fully notified when this happens.”*

The payoff rules were also slightly different in Study 2 (as explained on Study 2’s Methods section). The exact text in the instructions explaining the payoff rule was:

*“Your payoff from the current experiment will comprise of a fixed amount of 0.85£ for your participation, promised upon completion. Besides this amount, you will be paid a bonus payment comprising of an endowment of 0.1£ + your outcome in one randomly selected trial with a conversion rate of 1 point = 0.05£.”*

# Supplementary Appendix B

*Analysis of Game 1*, *Study 1.*


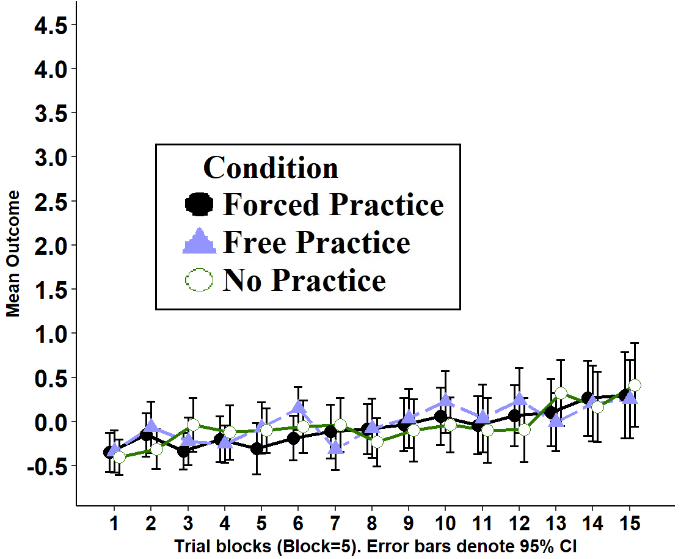

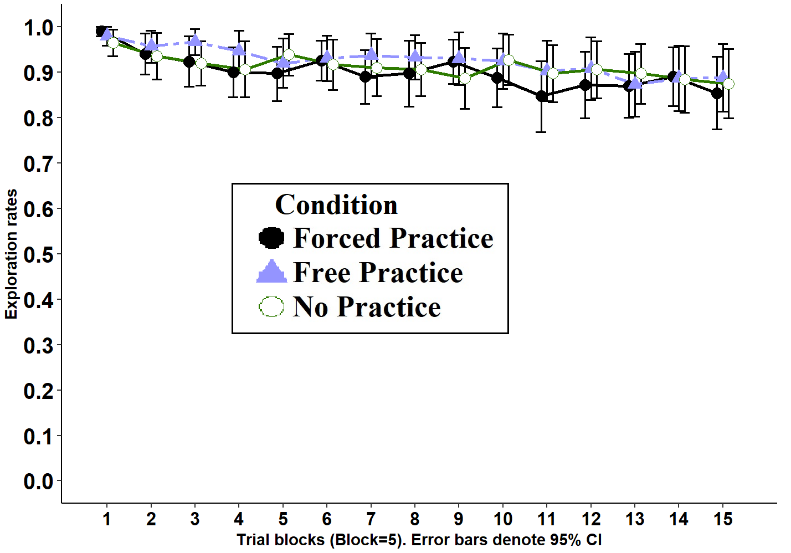


***Figure SA1.*** Left panel: Mean outcome in trial blocks (each block equals 5 trials) in Game 1, across the three Conditions of Study 1. Right panel: Mean exploration rates (choice rate of unfamiliar keys) in Game 1, across the three Conditions of Study 1.

*Analysis of Game 1*, *Study 2.*


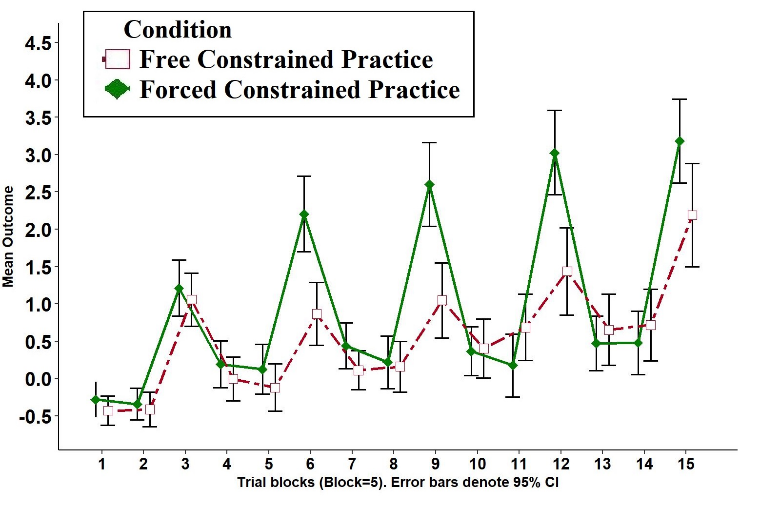

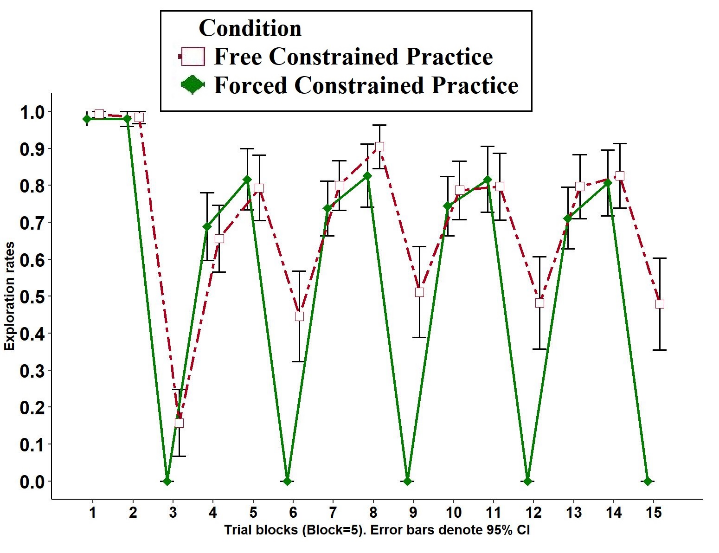


***Figure SA2.*** Left panel: Mean outcome in trial blocks (each block equals 5 trials) in Game 1, across the two Conditions of Study 2. Right panel: Mean exploration rates (choice rate of unfamiliar keys) in Game 1, across the two Conditions of Study 2. In this study, during the practice phase participants were constrained on every third trial block to only *exploit* familiar keys.

#
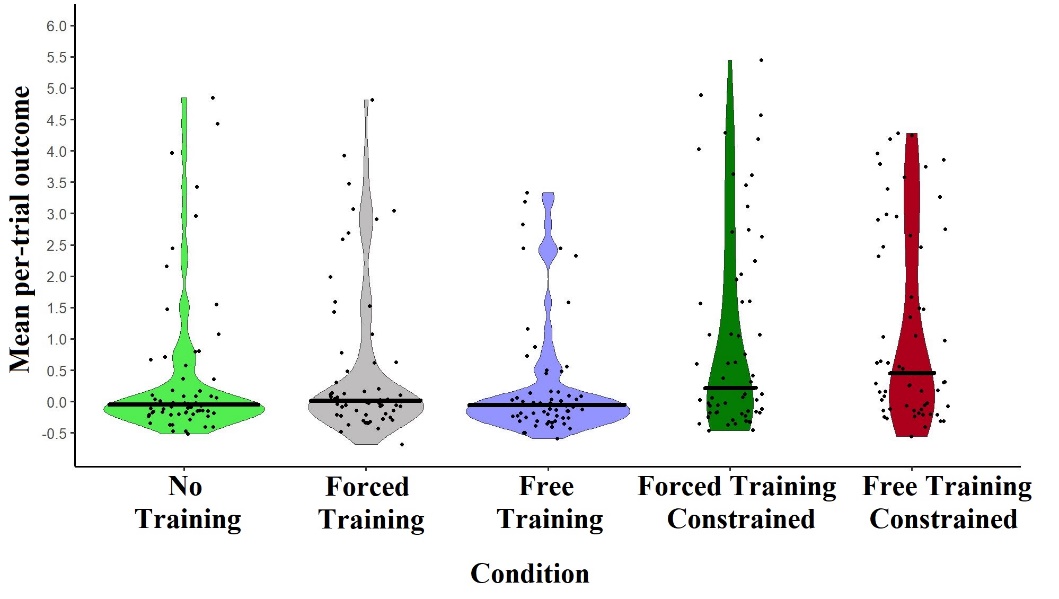
Supplementary Appendix C

***Figure SA1.*** Violin plots presenting the mean per-trial outcome for each individual participant in each of the five conditions of Studies 1 and 2. Each dot presents the mean outcome per trial of one individual participants in each condition. Black horizontal lines present the median outcome per individual participant.
